# Supplementary material for: Control of a gene transfer agent cluster in Caulobacter crescentus by transcriptional activation and anti-termination
Source: Nat Commun. 2024 Jun 4;15:4749. doi: 10.1038/s41467-024-49114-2 (PMC11150451; doi:10.1038/s41467-024-49114-2)
Supplement: Supplementary file 1 — Supplementary Information [file 41467_2024_49114_MOESM1_ESM.pdf]

SUPPLEMENTARY INFORMATION

Control of a gene transfer agent cluster in *Caulobacter crescentus* by transcriptional activation and anti-termination

*Ngat T. Tran & Tung B.K. Le**

Department of Molecular Microbiology

John Innes Centre, Norwich, NR4 7UH, United Kingdom

Correspondence: tung.le@jic.ac.uk

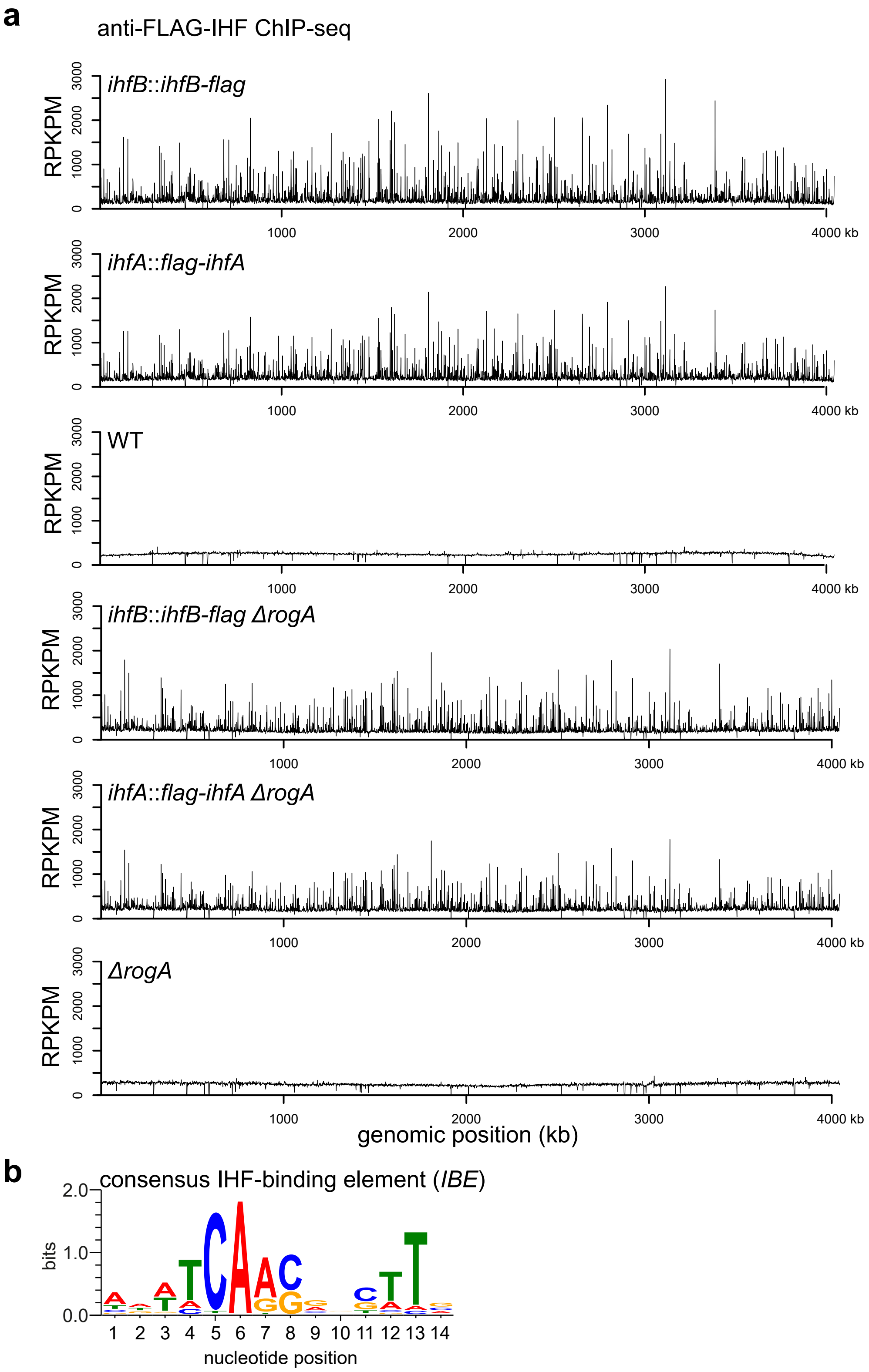

Supplementary Fig. 1. IHF binding on *C. crescentus* chromosome is independent of RogA. a anti-FLAG ChIP-seq profiles show the enrichment of FLAG-tagged IHFα and FLAG-tagged IHFβ on the chromosomes of either GTA-off WT or GTA-on Δ*rogA C. crescentus* cells in stationary phase. Profiles were plotted with the x-axis representing genomic positions and the y-axis representing the number of reads per kilobase pair per million mapped reads (RPKPM). ChIP-seq experiments were performed twice using biological replicates, and a representative profile is shown. **b** The consensus IHF-binding element (IHF), constructed from the hundred most enriched sites in anti-FLAG-IHF ChIP-seq datasets.

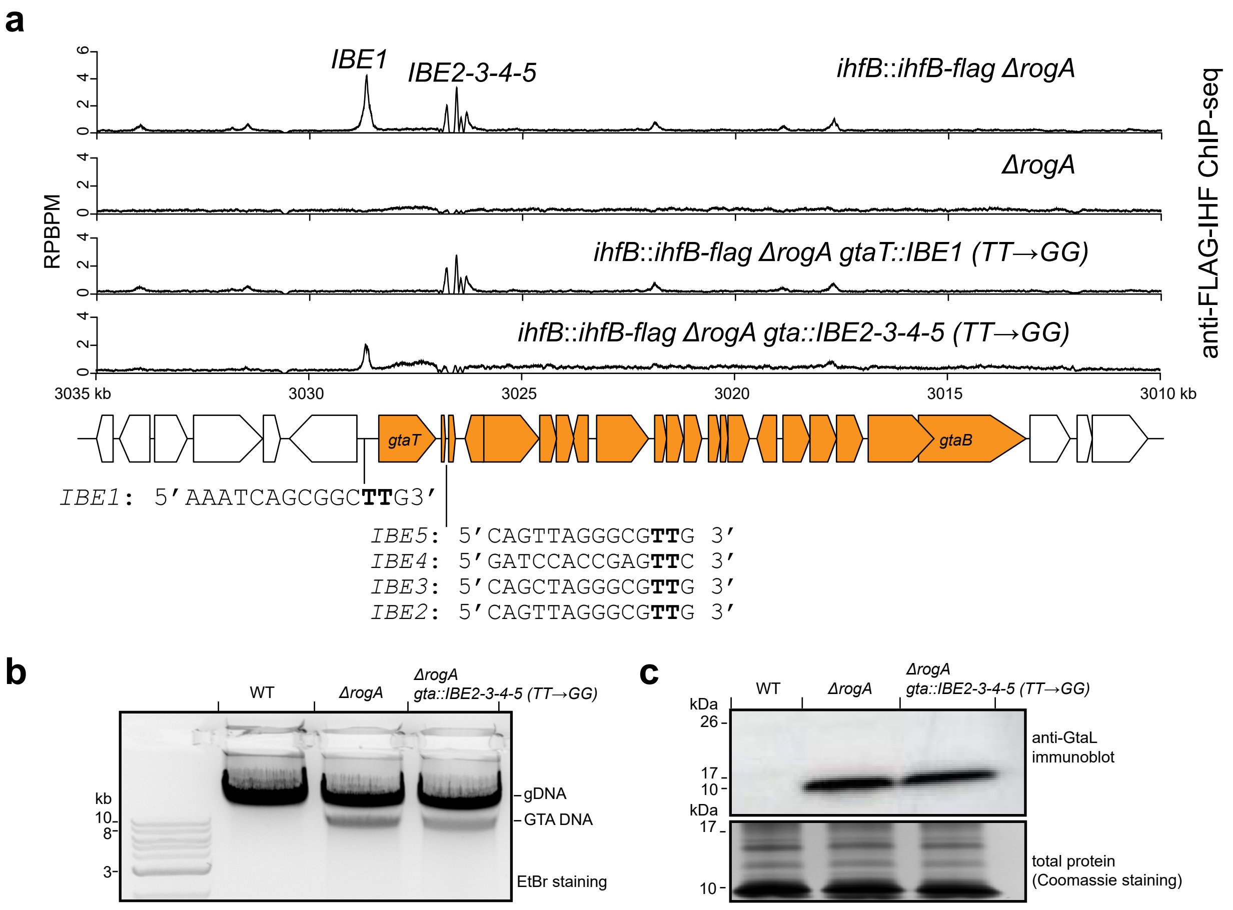

Supplementary Fig. 2. Four IHF binding elements (IBE 2-3-4-5) play no or little role in the transcriptional activation of the GTA main gene cluster. a anti-FLAG ChIP-seq profiles show the enrichment of FLAG-tagged IHFβ in different genetic backgrounds. Profiles were plotted with the x-axis representing genomic positions and the y-axis representing the number of reads per base pair per million mapped reads (RPBPM). The positions and sequences of identified IHF-binding elements (IBE) are shown beneath the schematic diagram of the GTA gene cluster. Note that the main GTA cluster is on the minus strand, but was inverted to run from left to right for a presentation purpose only. ChIP-seq experiments were performed twice using biological replicates, and a representative profile is shown. MACS2-identified ChIP-seq peaks above IBE 1, 2, 3, and 6 have Poisson distribution -$\log_{10}$(p value) and false discovery rate -$\log_{10}$(q value) > 1000 in both replicates. ChIP-seq peaks above IBE 4 and 5 were not reliably detected by MACS2 but have recognizable IHF-binding motif and were further characterized here. **b** Total DNA extraction from indicated strains grown up to a stationary phase. Experiments were performed at least twice, and a representative image is shown. **c** Immunoblot of total cell lysates of indicated strains using a polyclonal anti-GtaL (GTA head-tail connector protein) antibody. A separate Coomassie-stained SDS-PAGE was loaded with the same volume of samples to serve as a loading control. Immunoblots were performed at least twice, a representative image is shown. Source data are provided as a Source Data file for (**b-c**).

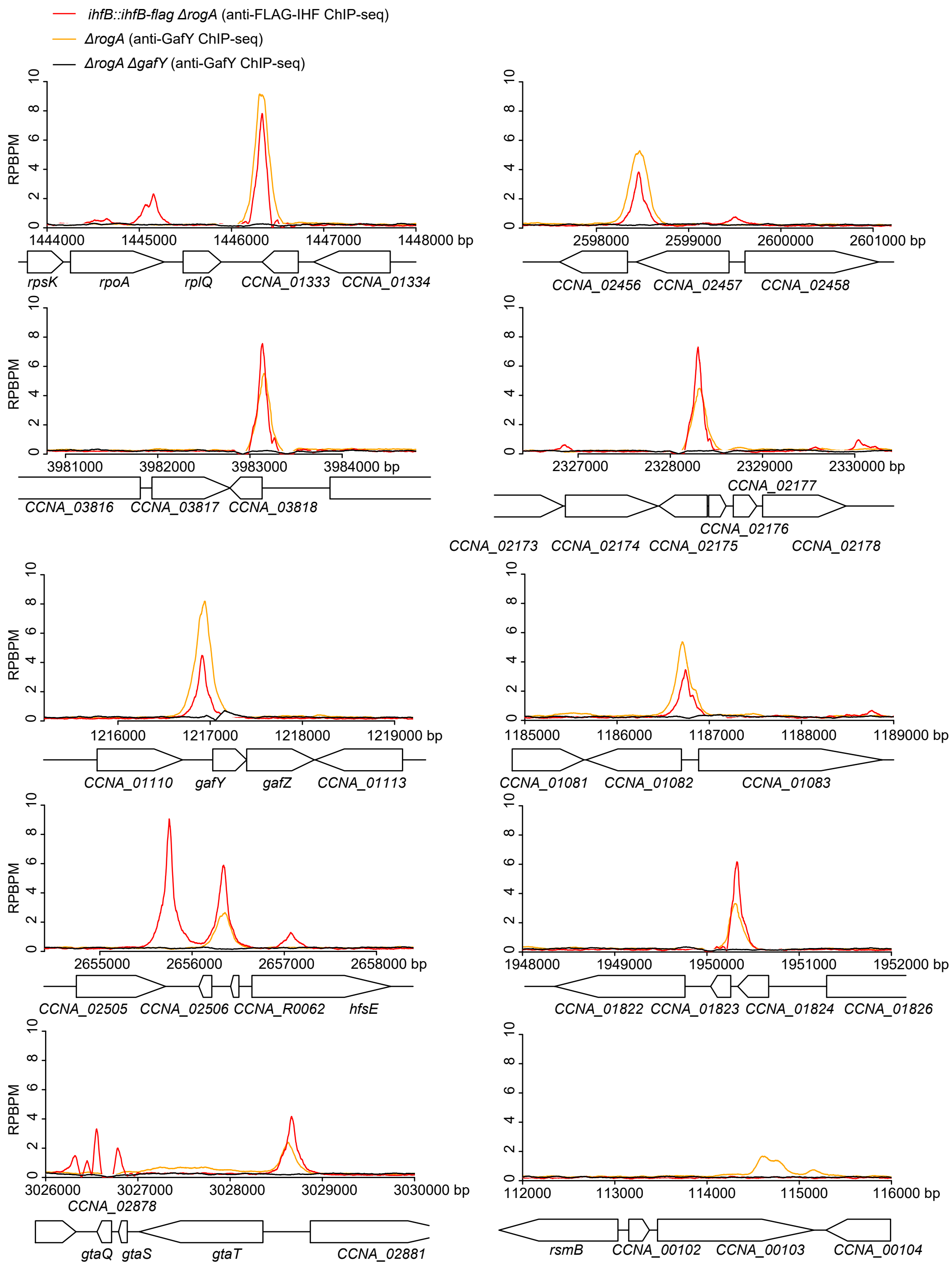

Supplementary Fig. 3. IHF binds to GafY-regulated promoters. anti-FLAG and anti-GafY ChIP-seq profiles show the enrichment of FLAG-tagged IHF (red), GafY (orange), and a negative control (black), respectively, at the ten most GafY-enriched regions. Profiles were plotted with the x-axis representing genomic positions and the y-axis representing the number of reads per base pair per million mapped reads (RPBPM). ChIP-seq experiments were performed twice using biological replicates, and a representative profile is shown.

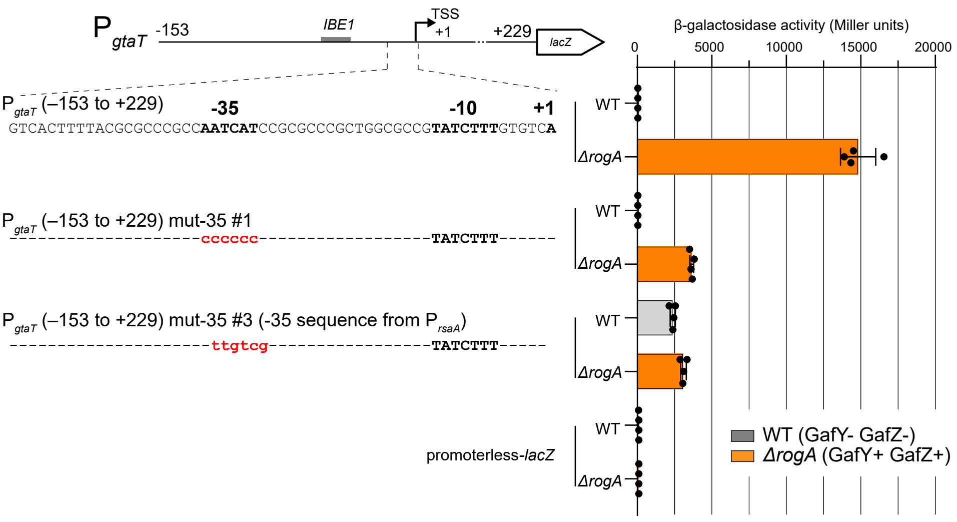

Supplementary Fig. 4. Substituting the -35 region of P$_{gtaT}$ by a near consensus -35 region from P$_{rsaA}$ caused constitutive expression. β-galactosidase activity (in Miller units) of the indicated promoter-*lacZ* reporter constructs in two *C. crescentus* genetic backgrounds (WT (grey) vs Δ*rogA* (orange)). The upstream region of *gtaT*, from -153 to +229 relative to the +1 TSS, was fused to *lacZ*. The -10 and -35 elements were mutated as indicated (red) on the schematic diagram. Cells containing an empty (promoterless) *lacZ* reporter plasmid served as a negative control. Values and error bars indicate mean ± standard deviations from four replicates. Source data are provided as a Source Data file.

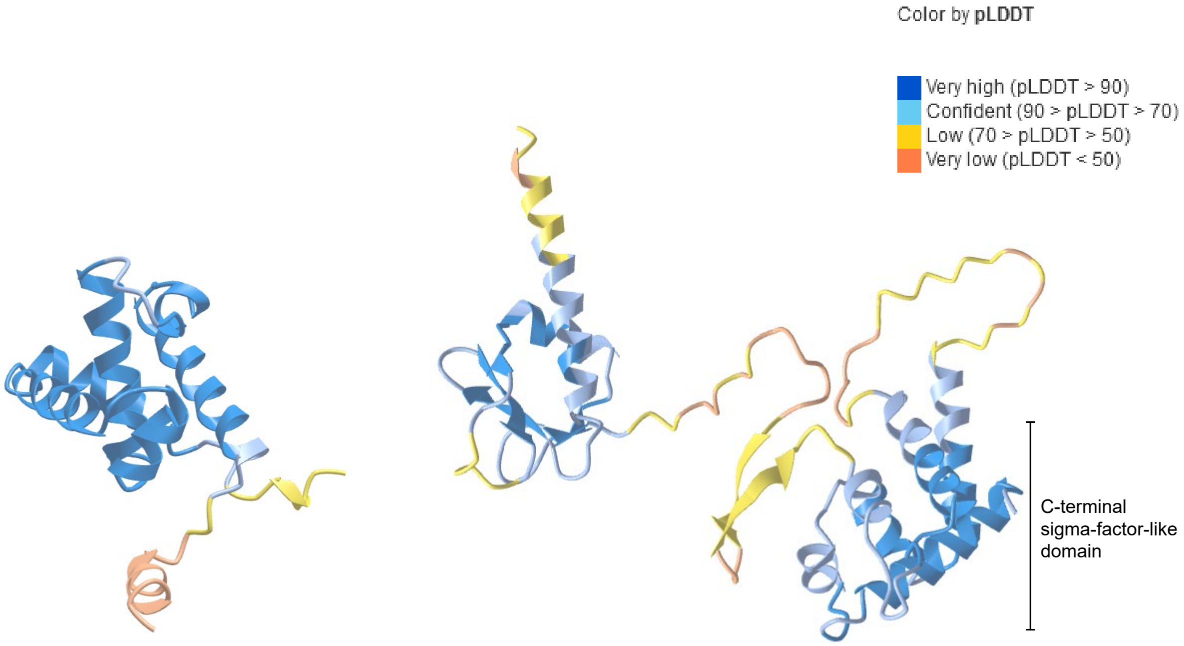

AlphaFold2-predicted structure of GafY

AlphaFold2-predicted structure of GafZ

Supplementary Fig. 5. AlphaFold-2 predicted structures of GafY (left panel) and GafZ (right panel). Predicted structures were colored according to their predicted local distance difference test scores (pLDDT). Source data are provided as a Source Data file.

a

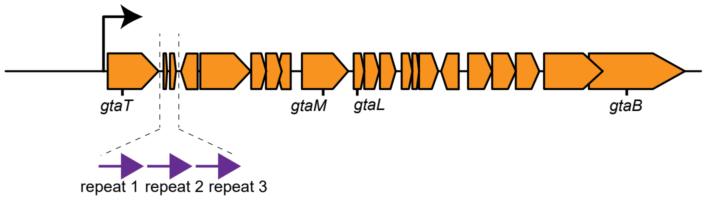


```
repeat3      CCCGCAGCTAGGGCGT-TGAAGAGGCGCGTGATTTCTCCTCCCCCTCTGGGGGAGGGGGA 59
repeat1      ------------------GGGCGGCCGAAGTAACTCTCCTCCCCCTCTGGGGGAGGGGGA 42
repeat2      CCCGCAGTTAGGGCGTTGAAGAGGCCGACTTATTTCTCCTCCCCCTCTGGGGGAGGGGGA 60
                                    * **    *  **************************

repeat3      CCAGCGAAGCTGGTGGAGGGGCCCGCTGCGGTGGCGCTACATCCTGTGGCCGGCCGATCC 119
repeat1      CCAGCGAAGCTGGTGGAGGGGCCCGCTGCGGTGGCGCTACATCCTGTGGCCGGCGGATCA 102
repeat2      CCAGCGAAGCTGGTGGAGGGGGCCTGTGCGGTGGCGTTACATCCTGTGGCCGGCGGATCA 120
             ********************* **  ********** ***************** **** 

repeat3      ACCGAGTTCTGAGATCGCCGGAGGGGCTATGCCGTTCGGCTGGCCCCTCCACCGGCGTTC 179
repeat1      GCCGAGTTCTGAAATCGCCGGAGGGGCTATGCCGTTCGGCTGGCCCCTCCACCGCCGTTC 162
repeat2      GCCGAGTTCTGAAATCGCCGGAGGGGCTATGCCGTTCGGCTGGCCCCTCCACCGGCGTTC 180
              *********** ***************************************** *****

repeat3      GCCGGTCCCCCTCCCCCAGAGGGGGAGGAGAGGTGTTCGCCGGGGCAAATTTTCCGCAGT 239
repeat1      GGCGGTCCCCCTCCCCCGATGGGGGAGGAGAGGTGTTCGCCGGGGGCAAATCT------- 215
repeat2      GCCGGTCCCCCTCCCCCAGAGGGGGAGGAGAGGTGTTCGCCGGGGCAAAATCT------- 233
             * ***************   *************************  ** * *       
```

b

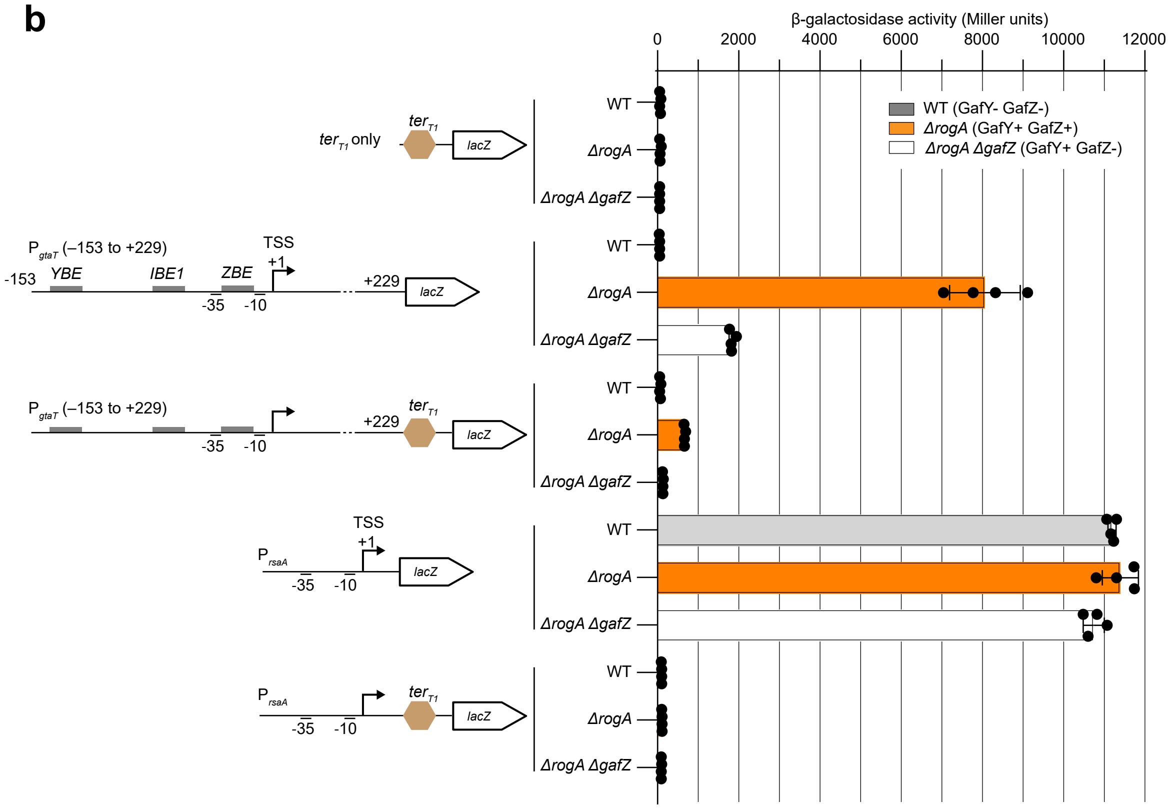

Supplementary Fig. 6. GafYZ allows RNAP to bypass a putative transcription terminator (*ter$_{GTA}$*) or an *E. coli rrnB* T1 transcription terminator (*ter$_{T1}$*) located downstream of *gtaT*. a Sequence alignment of three direct repeats (purple arrows) that constitute the putative terminator of the GTA cluster (*ter$_{GTA}$*). **b** β-galactosidase activity (in Miller units) of indicated promoter-*lacZ* reporter constructs in three *C. crescentus* genetic backgrounds (WT (grey) vs Δ*rogA* (orange) vs. Δ*rogA*Δ*gafZ* (white)). The upstream region of *gtaT*, from -153 to +229 relative to the +1 TSS, was fused to *lacZ*, with or without the *E. coli rrnB* T1 transcription terminator (*ter$_{T1}$*, light brown hexagon). Cells containing an empty (promoterless) *lacZ* reporter plasmid or plasmid with only *ter$_{T1}$* fused to *lacZ* served as negative controls. Values and error bars indicate mean ± standard deviations from four replicates. Source data are provided as a Source Data file for (**b**).

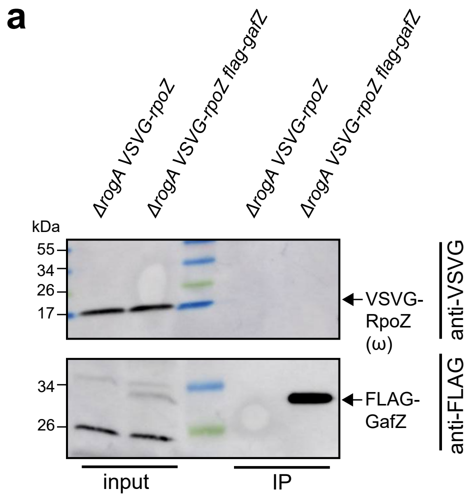

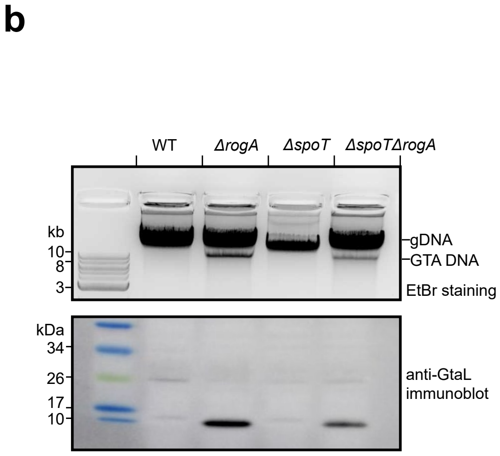

Supplementary Fig. 7. *C. crescentus* GafZ did not co-immunoprecipitate with *C. crescentus* RpoZ (ω subunit of RNA polymerase). a Immunoblots analysis of co-immunoprecipitation of FLAG-tagged GafZ. The positions of bands corresponding to VSVG-tagged RpoZ and FLAG-tagged GafZ are indicated with arrows. **b** Deletion of *spoT* did not eliminate GTA synthesis in *C. crescentus*. (top panel) Total DNA extraction from indicated strains grown up to a stationary phase. Total DNA was purified and separated by electrophoresis on a 1% agarose gel which was stained with ethidium bromide (EtBr) for DNA. Experiments were performed at least twice, and a representative image is shown. (bottom panel) Immunoblot of total cell lysates of indicated strains using a polyclonal anti-GtaL (GTA head-tail connector protein) antibody. Immunoblots were performed at least twice, and a representative image is shown. Source data are provided as a Source Data file for (**a-b**).

```
GafA      --MKT---------------------------------------MQDRESLPDWLPDHARLY  21
GafY      MLVQALEDIWAITPDPRRDRLTVGFVTHLVSLATGVPAVEIATPKRVSHTAVRARQLAIY  60
            :::                                       .* *       :  :*
GafA      LRHVEEGVPIRQLARAEGCHASTILRRVRRIEQRRDDPLVDEALTRLGRFAAAASAAPPR  81
GafY      LTHITLHWPLARVAFAFGRDRTTCGHACRKIEDLREDEAFDRRLCELEAC---LRQAPSH  117
          * *:    *: ::* * * . :*  :  *:**: *:*  .*. * .*         ** :
GafA      EDDPAMTAPIRPTAPQACPEAAEDPDSPDIATLSREGRRVLRRLAEPGALLIIAPDMEKA  141
GafY      AQ----------------------------------------------------------  119
           :
GafA      VVLRGTVRTAVVAREVAQGFALNGWILVQHSGRVTSYELSATGRAALKRLLEAEALTAGR  201
GafY      ------------------------------------------------------------  119

GafA      DPATAADNPHADRHRDWGERTVNEGQGRVTRMRMNLAESPLGVLARRRDSDGRPFLSPDL  261
GafY      ------DLP---------------------------------------------------  122
                * *
GafA      VAAGERLREDFELAQMGPRVAQNWERFMTGGARGQYRPELGHGGPGGSDRARERVAAALC  321
GafY      ------------------------------------------------------------  122

GafA      DLGPGLGDMVLRCCCFLEGLETAEKRMGWSARSGKIVLRIALMRLKRHYDETYGGAAPLI  381
GafY      ------------------------------------------------------------  122

GafA      G     382
GafY      -     122
- - - - - - - - - - - - - - - - - - - - - - - - - - - - - - - - - - - - 

GafA      MKTMQDRESLPDWLPDHARLYLRHVEEGVPIRQLARAEGCHASTILRRVRRIEQRRDDPL  60
GafZ      ------------------------------------------------------------  0

GafA      VDEALTRLGRFAAAASAAPPREDDPAMTAPIRPTAPQACPEAAEDPDSPDIATLSREGRR  120
GafZ      --------------------------MTGGLQI-----HFEA--------ALDAERRAER  21
                                    **. ::        **            .*...*
GafA      VLRRLAEPGALLIIAPDMEKAVVLRGTVR------------TAVVAREV-AQGFALNGWI  167
GafZ      AMRLLARPGAVIEPHG--GGYGVRLGASRRRCVMLTLDEATFAVLAREATLKPRREGGWT  79
          .:* **.***::          *  *: *             **:***.  :    .**
GafA      LVQHSGRVTSYELSATGRAALKRLLEAEALTAGRDPATAADNPHADRHRDWGERTVNEGQ  227
GafZ      MVARPE-------------------------------VAAVPPP-GR-PGVIEASVETA-  105
          :* :                                 .**  *  .*  .  * :*: .
GafA      GRVTRMRMNLAESPLGVLARRRDSDGRPFLSPDLVAAGERLREDFELAQMGPRVAQNWER  287
GafZ      --DGVVRRNLGESPIAWLARRRDSHGRPWLAPAEIAAAERLREEFESLGTLGRMTMRWDA  163
               :* **.***:. *******.***:*:*  :**.*****:**      *:: .*:
GafA      FMTGGA-RGQYRPELGHGGPGGSDRARERVAAALCDLGPGLGDMVLRCCCFLEGLETAEK  346
GafZ      TPRVDGGQTVLA------PAERDHATRQRIARALTAVGPGLRDILERVCLMGSALEAAED  217
              .. :              .. :*:*:* **  :**** *:: * * : ..**:**.
GafA      RMGWSARSGKIVLRIALMRLKRHYDETYGGAAPLIG       382
GafZ      SLKLPRRAGKTVLKLALQALARHYRMA---------       244
           :    *:** **::**  * ***  :
```

Supplementary Fig. 8. Sequence alignment of *R. capsulatus* GafA to *C. crescentus* GafY and GafZ. The N-terminal domain, the central domain, and the C-terminal domain of GafA are colored in dark green, blue, and cyan, respectively. The sequence alignment shows poor amino acid similarity for the central region of *R. capsulatus* GafA and the N-terminal side of *C. crescentus* GafZ (shaded box).

SUPPLEMENTARY METHODS

Construction of plasmids and strains

pNPTS138::gtaM-lacA

The left flanking region (~500bp) of the insertion site, *lacA*, and the right flanking region (~500bp) of the insertion site were amplified by PCR using TLO3300+TLO3301, TLO3302+TLOO3303, TLO3304+TLOO3305 respectively. PCR products were purified from gel and assembled to BamHI-HindIII-cut pNPTS138 using 2x Gibson mastermix following the manufacturer's instruction. The Gibson reaction was used to transform chemically competent *E. coli* DH5α cells. The resulting plasmid was sequence verified by Sanger sequencing and used to transform *ΔlacA* competent cells to construct NTS2261.

pNPTS138::flag-ihfA

The left and right flanking regions (~500bp) of the insertion site were amplified by PCR using oligo NTO2830+NTO2831, NTO2832+NTO2833. PCR products were purified from gel and assembled to BamHI-HindIII-cut pNPTS138 using 2x Gibson mastermix following the manufacturer's instruction. The Gibson reaction was used to transform chemically competent *E. coli* DH5α cells. The resulting plasmid was sequence verified by Sanger sequencing and used to transform *C. crescentus* CB15N to make NTS2689.

pNPTS138::ihfB-flag

The left and right flanking regions (~500bp) of the insertion site were amplified by PCR using oligo using oligo NTO2745+NTO2746, NTO2747+NTO2748. PCR products were purified from gel and assembled to BamHI-HindIII-cut pNPTS138 using 2x Gibson mastermix following the manufacturer's instruction. The Gibson reaction was used to transform chemically competent *E. coli* DH5α cells. The resulting plasmid was sequence verified by Sanger sequencing and used to (i) transform *C. crescentus* CB15N to make NTS2632, (ii) transform NTS2724 to make NTS2745, and (iii) transform NTS2729 to make NTS2747.

pNPTS138::ΔihfB

The left and right flanking regions (~500bp) of the deletion site were amplified by PCR using oligo NTO2781+NTO2782, NTO2783+NTO2784. PCR products were purified from gel and assembled to BamHI-HindIII-cut pNPTS138 using 2x Gibson mastermix following the manufacturer's instruction. The Gibson reaction was used to transform chemically competent *E. coli* DH5α cells. The resulting plasmid

was sequence verified by Sanger sequencing and used to transform *C. crescentus* CB15N to make NTS2608.

pNPTS138::ΔihfA

The left and right flanking regions (~500bp) of the deletion site were amplified by PCR using oligo NTO2799+NTO2800, NTO2801+NTO2802. PCR products were purified from gel and assembled to BamHI-HindIII-cut pNPTS138 using 2x Gibson mastermix following the manufacturer's instruction. The Gibson reaction was used to transform chemically competent *E. coli* DH5α cells. The resulting plasmid was sequence verified by Sanger sequencing and used to transform *C. crescentus* CB15N to make NTS2688.

pXYFPC-1::ihfA

ihfA orf was amplified by PCR from *C. crescentus* genomic DNA using NTO2845+NTO2846. PCR products were purified from gel and assembled to NdeI-NheI-cut pXYFPC-1 using 2x Gibson mastermix following the manufacturer's instructions. The Gibson reaction was used to transform chemically competent *E. coli* DH5α cells. The resulting plasmid was sequence verified by Sanger sequencing and used to transform NTS2692.

pXYFPC-1::ihfB

ihfB orf was amplified by PCR from *C. crescentus* genomic DNA using NTO2767+NTO2768. PCR products were purified from gel and assembled to NdeI-NheI-cut pXYFPC-1 using 2x Gibson mastermix following the manufacturer's instructions. The Gibson reaction was used to transform chemically competent *E. coli* DH5α cells. The resulting plasmid was sequence verified by Sanger sequencing and used to transform NTS2609.

pNPTS138::ΔgtaT IBE1

To delete of 51bp spanning IHF binding element located in the promoter region of *gtaT* (*CCNA_02880*), the left and right flanking regions (~500bp) of the deletion site were amplified by PCR using oligo NTO2230+NTO2753, NTO2754+NTO2755. PCR products were purified from gel and assembled to BamHI-HindIII-cut pNPTS138 using 2x Gibson mastermix following the manufacturer's instruction. The Gibson reaction was used to transform chemically competent *E. coli* DH5α cells. The resulting plasmid was sequence verified by Sanger sequencing and used to transform *C. crescentus* CB15N to make NTS2623.

pNPTS138::gtaT IBE1 (TT to GG)

To change IHF binding element (IBE) in the promoter region of *gtaT* from TT to GG, the left and right flanking regions (~500bp) of the insertion site were amplified by PCR using oligo NTO2230+NTO2863, NTO2864+NTO2755. PCR products were purified from gel and assembled to BamHI-HindIII-cut pNPTS138 using 2x Gibson mastermix following the manufacturer's instruction. The Gibson reaction was used to transform chemically competent *E. coli* DH5α cells. The resulting plasmid was sequence verified by Sanger sequencing and used to transform NTS2623 to make NTS2724.

pNPTS138::ΔgafYZ IBE6

To delete 42-bp spanning IHF binding element located in the promoter region of *gafY*, the left and right flanking regions (~500bp) of the deletion site were amplified by PCR using oligo NTO2749+NTO2750, NTO2751+NTO2752. PCR products were purified from gel and assembled to BamHI-HindIII-cut pNPTS138 using 2x Gibson mastermix following the manufacturer's instruction. The Gibson reaction was used to transform chemically competent *E. coli* DH5α cells. The resulting plasmid was sequence verified by Sanger sequencing and used to transform *C. crescentus* CB15N to make NTS2626.

pNPTS138::gafYZ IBE6 (TT to GG)

To change IHF binding element in the promoter region of *gafY* from TT to GG, the flanking regions of the insertion site were amplified by PCR using oligo 2749+2865, 2866+2752. PCR products were purified from gel and assembled to BamHI-HindIII-cut pNPTS138 using 2x Gibson mastermix following the manufacturer's instruction. The Gibson reaction was used to transform chemically competent *E. coli* DH5α cells. The resulting plasmid was sequence verified by Sanger sequencing and used to transform NTS2626 to make NTS2729.

pNPTS138::Δihf500

To delete 500bp spanning 4 IHF binding elements (IBE2-3-4-5) located inside the phage cluster, the left and right flanking regions (~500bp) of the deletion site were amplified by PCR using oligo NTO2888+NTO2889, NTO2890+NTO2891. PCR products were purified from gel and assembled to BamHI-HindIII-cut pNPTS138 using 2x Gibson mastermix following the manufacturer's instruction. The Gibson reaction was used to transform chemically competent *E. coli* DH5α cells. The resulting plasmid was sequence verified by Sanger sequencing and used to transform CB15N to make NTS2755.

pNPTS138::ihfall4GG

To change 4 IHF binding elements inside the phage cluster, the flanking regions of the insertion site were amplified by PCR using oligo NTO2888+NTO2892, NTO2893+NTO2929, NTO2932+NTO2935, NTO2934+NTO2905, NTO2899+NTO2891. PCR products were purified from gel and assembled to BamHI-HindIII-cut pNPTS138 using 2x Gibson mastermix following the manufacturer's instruction. The Gibson reaction was used to transform chemically competent *E. coli* DH5α cells. The resulting plasmid was sequence verified by Plasmidsaurus and used to transform NTS2755 to make NTS2986.

pNPTS138::nusG-vsvg

To insert a VSVG tag at the C terminus of NusG, the flanking regions (~500bp) of the insertion site were amplified by PCR using oligo NTO2923+NTO2924, NTO2925+NTO2926. PCR products were purified from gel and assembled to BamHI-HindIII-cut pNPTS138 using 2x Gibson mastermix following the manufacturer's instruction. The Gibson reaction was used to transform chemically competent *E. coli* DH5α cells. The resulting plasmid was sequence verified by Sanger sequencing and used to transform CB15N to make NTS2758, and NTS2481 to make NTS2759, respectively.

pNPTS138::vsvg-nusA

To insert a VSVG tag at the N terminus of NusA, the flanking regions (~500bp) of the insertion site were amplified by PCR using oligo NTO4041+NTO4042, NTO4043+NTO4044. PCR products were purified from gel and assembled to BamHI-HindIII-cut pNPTS138 using 2x Gibson mastermix following the manufacturer's instruction. The Gibson reaction was used to transform chemically competent *E. coli* DH5α cells. The resulting plasmid was sequence verified by Sanger sequencing and used to transform CB15N to make NTS2897, and NTS2481 to make NTS2863, respectively.

pNPTS138::vsvg-nusE

To insert a VSVG tag at the N terminus of NusE, the flanking regions (~500bp) of the insertion site were amplified by PCR using oligo NTO4037+NTO4038, NTO4039+NTO4040. PCR products were purified from gel and assembled to BamHI-HindIII-cut pNPTS138 using 2x Gibson mastermix following the manufacturer's instruction. The Gibson reaction was used to transform chemically competent *E. coli* DH5α cells. The resulting plasmid was sequence verified by Sanger sequencing and used to transform CB15N to make NTS2901, and NTS2481 to make NTS2857, respectively.

pNPTS138::vsvg-rpoZ

To insert a VSVG tag at the N-terminus of RpoZ, the flanking regions (~500bp) of the insertion site were amplified by PCR using oligos NTO2785 and NTO2786, NTO2787 and NTO2788. PCR products were

purified from gel and assembled to BamHI-HindIII-cut pNPTS138 using 2x Gibson mastermix following the manufacturer's instruction. The Gibson reaction was used to transform chemically competent *E. coli* DH5α cells. The resulting plasmid was sequence verified by Sanger sequencing and used to transform CB15N to make NTS2662, and to transform NTS2489 to make NTS2681.

pNPTS138:: *ΔspoT:: tetracyclineR*

The left and right flanking regions (~500bp) of the deletion site were amplified by PCR using oligos NTO2327 and NTO2328, NTO2329 and NTO2330, and the tetracycline resistance cassette was amplified by PCR using oligos NTO2303 and NTO2304. PCR products were purified from gel and assembled to BamHI-HindIII-cut pNPTS138 using 2x Gibson mastermix following the manufacturer's instruction. The Gibson reaction was used to transform chemically competent *E. coli* DH5α cells. The resulting plasmid was sequence verified by Sanger sequencing and used to transform *C. crescentus* CB15N to make NTS2374

pNPTS138::flag-rpoD

To insert a FLAG tag at the N terminus of RpoD, the flanking regions (~500bp) of the insertion site were amplified by PCR using oligo NTO4268+NTO4269, NTO4270+NTO4271. PCR products were purified from gel and assembled to BamHI-HindIII-cut pNPTS138 using 2x Gibson mastermix following the manufacturer's instruction. The Gibson reaction was used to transform chemically competent *E. coli* DH5α cells. The resulting plasmid was sequence verified by Sanger sequencing and used to transform CB15N to make NTS4195.

pNTP2846

To remove 2 BamHI sites from pXYFPC-1, using pXYFPC-1 as template, DNA fragments were amplified by PCR using NTO4188+NTO4191, NTO4189+NTO4190. PCR products were purified from gel and assembled using 2x Gibson mastermix following the manufacturer's instructions. The Gibson reaction was used to transform chemically competent *E. coli* DH5α cells. The resulting plasmid was sequence verified by Sanger sequencing.

pNTP2864

To remove the HindIII site from pNTP2846, using pNTP2846 as template, DNA fragments were amplified by PCR using NTO4192+NTO4195, NTO4193+NTO4194. PCR products were purified from gel and assembled using 2x Gibson mastermix following the manufacturer's instructions. The Gibson

reaction was used to transform chemically competent *E. coli* DH5α cells. The resulting plasmid was sequence verified by Sanger sequencing which is devoid of BamHI and HindIII sites.

pRlacZ290:: PgtaT (-153 to +229)

DNA fragment from –153 to +229 of GTA cluster promoter was amplified by PCR from genomic DNA using NTO4085+NTO4101. PCR products were purified from gel and assembled to EcoRI-KpnI-cut pRlacZ290 using 2x Gibson mastermix following the manufacturer's instruction. The Gibson reaction was used to transform chemically competent *E. coli* DH5α cells. The resulting plasmid was sequence verified by Sanger sequencing.

pNTP2864:: PgtaT (-153 to +229)

DNA fragment from –153 to +229 of GTA cluster promoter together with *lacZ* in pRlacZ290:: PgtaT (-153 to +229) was amplified by PCR using NTO4196+NTO4197. PCR products were purified from gel and assembled to NdeI-NheI-cut pNTP2864 using 2x Gibson mastermix following the manufacturer's instruction. The Gibson reaction was used to transform chemically competent *E. coli* DH5α cells. The resulting plasmid was sequence verified by whole-plasmid sequencing (Plasmidsaurus).

pNPTS138:: PgtaT (-113 to +229)

DNA fragment from –113 to +229 of GTA cluster promoter was amplified by PCR from genomic DNA using NTO4259+NTO4185. PCR products were purified from gel and assembled to BamHI-HindIII-cut pNPTS138 using 2x Gibson mastermix following the manufacturer's instruction. The Gibson reaction was used to transform chemically competent *E. coli* DH5α cells. The resulting plasmid was sequence verified by Sanger sequencing.

pNTP2864:: PgtaT (-113 to +229)

DNA fragment from –113 to +229 of GTA cluster promoter was removed from pNPTS138:: PgtaT (-113 to +229) and ligated to EcoRI-KpnI-cut pNTP2864:: PgtaT (-153 to +229). The ligation mixture was used to transform chemically competent *E. coli* DH5α cells. The resulting plasmid was verified by restriction digestion.

pNPTS138:: PgtaT (-56 to +229)

DNA fragment from –56 to +229 of GTA cluster promoter was amplified by PCR from genomic DNA using NTO4369+NTO4185. PCR products were purified from gel and assembled to BamHI-HindIII-cut pNPTS138 using 2x Gibson mastermix following the manufacturer's instruction. The Gibson reaction

was used to transform chemically competent *E. coli* DH5α cells. The resulting plasmid was sequence verified by Sanger sequencing.

pNTP2864:: PgtaT (-56 to +229)

DNA fragment from –56 to +229 of GTA cluster promoter was removed from pNPTS138:: PgtaT (-56 to +229) and ligated to EcoRI-KpnI-cut pNTP2864:: PgtaT (-153 to +229). The ligation mixture was used to transform chemically competent *E. coli* DH5α cells. The resulting plasmid was verified by restriction digestion.

pNPTS138:: PgtaT (-143 to +229)

DNA fragment from –143 to +229 of GTA cluster promoter was amplified by PCR from genomic DNA using NTO4256+NTO4185. PCR products were purified from gel and assembled to BamHI-HindIII-cut pNPTS138 using 2x Gibson mastermix following the manufacturer's instruction. The Gibson reaction was used to transform chemically competent *E. coli* DH5α cells. The resulting plasmid was sequence verified by Sanger sequencing.

pNTP2864:: PgtaT (-143 to +229)

DNA fragment from –143 to +229 of GTA cluster promoter was removed from pNPTS138:: PgtaT (-143 to +229) and ligated to EcoRI-KpnI-cut pNTP2864:: PgtaT (-153 to +229). The ligation mixture was used to transform chemically competent *E. coli* DH5α cells. The resulting plasmid was verified by restriction digestion.

pNPTS138:: PgtaT (-133 to +229)

DNA fragment from –133 to +229 of GTA cluster promoter was amplified by PCR from genomic DNA using NTO4257+NTO4185. PCR products were purified from gel and assembled to BamHI-HindIII-cut pNPTS138 using 2x Gibson mastermix following the manufacturer's instruction. The Gibson reaction was used to transform chemically competent *E. coli* DH5α cells. The resulting plasmid was sequence verified by Sanger sequencing.

pNTP2864:: PgtaT (-133 to +229)

DNA fragment from –133 to +229 of GTA cluster promoter was removed from pNPTS138:: PgtaT (-133 to +229) and ligated to EcoRI-KpnI-cut pNTP2864:: PgtaT (-153 to +229). The ligation mixture was used to transform chemically competent *E. coli* DH5α cells. The resulting plasmid was verified by restriction digestion.

pNPTS138:: PgtaT (-123 to +229)

DNA fragment from –123 to +229 of GTA cluster promoter was amplified by PCR from genomic DNA using NTO4258+NTO4185. PCR products were purified from gel and assembled to BamHI-HindIII-cut pNPTS138 using 2x Gibson mastermix following the manufacturer's instruction. The Gibson reaction was used to transform chemically competent *E. coli* DH5α cells. The resulting plasmid was sequence verified by Sanger sequencing.

pNTP2864::PgtaT (-123 to +229)

DNA fragment from –123 to +229 of GTA cluster promoter was removed from pNPTS138:: PgtaT (-123 to +229) and ligated to EcoRI-KpnI-cut pNTP2864:: PgtaT (-153 to +229). The ligation mixture was used to transform chemically competent *E. coli* DH5α cells. The resulting plasmid was verified by restriction digestion.

pNTPS138:: Δ125bp (+90 to +214)

To delete 125bp in gtaT to find the correct start codon, the flanking regions (~500bp) of the deletion site were amplified by PCR using NTO2230+NTO2764, NTO2765+NTO2766. PCR products were purified from gel and assembled to BamHI-HindIII-cut pNPTS138 using 2x Gibson mastermix following the manufacturer's instruction. The Gibson reaction was used to transform chemically competent *E. coli* DH5α cells. The resulting plasmid was sequence verified by Sanger sequencing, and introduced into CB15N to make NTS2653.

pNTPS138:: Δ144bp (+230 to 373)

To delete 144bp in *gtaT* to find the correct start codon, the flanking regions (~500bp) of the deletion site were amplified by PCR using NTO2957+NTO2958, NTO2960+NTO2961. PCR products were purified from gel and assembled to BamHI-HindIII-cut pNPTS138 using 2x Gibson mastermix following the manufacturer's instruction. The Gibson reaction was used to transform chemically competent *E. coli* DH5α cells. The resulting plasmid was sequence verified by Sanger sequencing, and introduced into CB15N to make NTS2792.

pNPTS138::gtaT (ATG1 to TGA)

To mutagenize the 1st ATG codon to TGA, the flanking regions (~500bp) of the insertion site were amplified by PCR using NTO2957+NTO2962, NTO2963+NTO2961. PCR products were purified from gel and assembled to BamHI-HindIII-cut pNPTS138 using 2x Gibson mastermix following the

manufacturer's instruction. The Gibson reaction was used to transform chemically competent *E. coli* DH5α cells. The resulting plasmid was sequence verified by Sanger sequencing, and introduced into NTS2792 to make NTS2800.

pNPTS138:: gtaT (ATG2 to TGA)

To mutagenize the 2nd ATG codon to TGA, the flanking regions (~500bp) of the insertion site were amplified by PCR using NTO2957+NTO2964, NTO2965+NTO2961. PCR products were purified from gel and assembled to BamHI-HindIII-cut pNPTS138 using 2x Gibson mastermix following the manufacturer's instruction. The Gibson reaction was used to transform chemically competent *E. coli* DH5α cells. The resulting plasmid was sequence verified by Sanger sequencing, and introduced into NTS2792 to make NTS2801.

pNPTS138:: gtaT (ATG3 to TGA)

To mutagenize the 3rd ATG codon to TGA, the flanking regions (~500bp) of the insertion site were amplified by PCR using NTO2957+NTO2966, NTO2967+NTO2961. PCR products were purified from gel and assembled to BamHI-HindIII-cut pNPTS138 using 2x Gibson mastermix following the manufacturer's instruction. The Gibson reaction was used to transform chemically competent *E. coli* DH5α cells. The resulting plasmid was sequence verified by Sanger sequencing, and introduced into NTS2792 to make NTS2802.

pNPTS138::Δ336bp (-113 to +223)

To delete 336bp from the putative start codon TTG of gtaT to the putative RBS, the flanking regions (~500bp) of the deletion site were amplified by PCR using NTO2230+NTO2906, NTO2907+NTO2766. PCR products were purified from gel and assembled to BamHI-HindIII-cut pNPTS138 using 2x Gibson mastermix following the manufacturer's instruction. The Gibson reaction was used to transform chemically competent *E. coli* DH5α cells. The resulting plasmid was sequence verified by Sanger sequencing, and introduced into CB15N to make NTS2757.

pNPTS138::gtaT (TTG to CTG)

To change the annotated start codon of gtaT from TTG to CTG, the flanking regions (~500bp) of the insertion site were amplified by PCR using NTO2230+NTO2908, NTO2909+NTO2766. PCR products were purified from gel and assembled to BamHI-HindIII-cut pNPTS138 using 2x Gibson mastermix following the manufacturer's instruction. The Gibson reaction was used to transform chemically

competent *E. coli* DH5α cells. The resulting plasmid was sequence verified by Sanger sequencing, and introduced into NTS2757 to make NTS2767.

pNPTS138::ΔYBE

To delete GafY binding element (YBE) in GTA cluster promoter, the flanking regions (~500bp) of the deletion site were amplified by PCR using NTO2230+NTO2970, NTO2971+NTO2755. PCR products were purified from gel and assembled to BamHI-HindIII-cut pNPTS138 using 2x Gibson mastermix following the manufacturer's instruction. The Gibson reaction was used to transform chemically competent *E. coli* DH5α cells. The resulting plasmid was sequence verified by Sanger sequencing, and introduced into CB15N to make NTS2799.

pNPTS138:: YBE* (ACTATG to TGACCC)

To change GafY binding element in GTA cluster promoter from ACTATGGGGA to tgacccGGGA, the flanking regions (~500bp) of the insertion site were amplified by PCR using NTO2230+NTO4157, NTO4158+NTO2755. PCR products were purified from gel and assembled to BamHI-HindIII-cut pNPTS138 using 2x Gibson mastermix following the manufacturer's instruction. The Gibson reaction was used to transform chemically competent *E. coli* DH5α cells. The resulting plasmid was sequence verified by Sanger sequencing, and introduced into NTS2799 to make NTS2940.

pNPTS138::ΔGTA core promoter

To delete the core promoter of GTA cluster, the flanking regions (~500bp) of the deletion site were amplified by PCR using NTO2230+NTO2760, NTO2761+NTO2755. PCR products were purified from gel and assembled to BamHI-HindIII-cut pNPTS138 using 2x Gibson mastermix following the manufacturer's instruction. The Gibson reaction was used to transform chemically competent *E. coli* DH5α cells. The resulting plasmid was sequence verified by Sanger sequencing, and introduced into CB15N to make NTS2650.

pNPTS138::ZBE* (CTGGCGC to TTTTTTC)

To change GafZ binding element (ZBE) from GCGCCCGCTGGCGC to GCGCCCGTTTTTTC, the flanking regions (~500bp) of the insertion site were amplified by PCR using NTO2230+NTO4090, NTO4091+NTO2755. PCR products were purified from gel and assembled to BamHI-HindIII-cut pNPTS138 using 2x Gibson mastermix following the manufacturer's instruction. The Gibson reaction was used to transform chemically competent *E. coli* DH5α cells. The resulting plasmid was sequence

verified by Sanger sequencing, and introduced into NTS2650 to make NTS2955. pNPTS138::*flag-gafZ* was introduced into NTS2955 to make NTS3024.

pNPTS138::mut10-1

To change the -10 promoter sequence of GTA cluster promoter from TATCTTT to ccccccc, PCR amplifies fragments using NTO4184+NTO4174, NTO4175+NTO4185. PCR products were purified from gel and assembled to BamHI-HindIII-cut pNPTS138 using 2x Gibson mastermix following the manufacturer's instruction. The Gibson reaction was used to transform chemically competent *E. coli* DH5α cells. The resulting plasmid was sequence verified by Sanger sequencing.

pNTP2864::mut10-1

GTA cluster promoter with the -10 sequence changed from TATCTTT to ccccccc was removed from pNPTS138::mut10-1 as EcoRI-KpnI fragment and ligated to EcoRI-KpnI-cut pNTP2864:: PgtaT (-153 to +229) The ligation mixture was used to transform chemically competent *E. coli* DH5α cells. The resulting plasmid was verified by restriction digestion.

pNPTS138::mut10-2

To change the -10 promoter sequence of GTA cluster promoter from TATCTTTGT to TATCTcccc, PCR amplifies fragments using NTO4184+NTO4176, NTO4177+NTO4185. PCR products were purified from gel and assembled to BamHI-HindIII-cut pNPTS138 using 2x Gibson mastermix following the manufacturer's instruction. The Gibson reaction was used to transform chemically competent *E. coli* DH5α cells. The resulting plasmid was sequence verified by Sanger sequencing.

pNTP2864::mut10-2

GTA cluster promoter with -10 sequence changed from TATCTTTGT to TATCTcccc was removed from pNPTS138::mut10-2 as EcoRI-KpnI fragment and ligated to EcoRI-KpnI-cut pNTP2864:: PgtaT (-153 to +229). The ligation mixture was used to transform chemically competent *E. coli* DH5α cells. The resulting plasmid was verified by restriction digestion.

pNPTS138::mut35-1

To change the -35 promoter sequence of GTA cluster promoter from AATCAT to cccccc, PCR amplifies fragments using NTO4184+NTO4242, NTO4243+NTO4185. PCR products were purified from gel and assembled to BamHI-HindIII-cut pNPTS138 using 2x Gibson mastermix following the manufacturer's

instruction. The Gibson reaction was used to transform chemically competent *E. coli* DH5α cells. The resulting plasmid was sequence verified by Sanger sequencing.

pNTP2864::mut35-1

GTA cluster promoter with the -35 sequence changed from AATCAT to cccccc was removed from pNPTS138::mut35-1 as EcoRI-KpnI fragment and ligated to EcoRI-KpnI-cut pNTP2864:: PgtaT (-153 to +229). The ligation mixture was used to transform chemically competent *E. coli* DH5α cells. The resulting plasmid was sequence verified by Sanger sequencing.

pNPTS138::mut35-2

To change the -35 promoter sequence of GTA cluster promoter from AATCAT to AAgggT, PCR amplifies fragments using NTO4184+NTO4264, NTO4265+NTO4185. PCR products were purified from gel and assembled to BamHI-HindIII-cut pNPTS138 using 2x Gibson mastermix following the manufacturer's instruction. The Gibson reaction was used to transform chemically competent *E. coli* DH5α cells. The resulting plasmid was sequence verified by Sanger sequencing.

pNTP2864::mut35-2

GTA cluster promoter with the -35 sequence changed from AATCAT to AAgggT was removed from pNPTS138::mut35-2 as EcoRI-KpnI fragment and ligated to EcoRI-KpnI-cut pNTP2864:: PgtaT (-153 to +229). The ligation mixture was used to transform chemically competent *E. coli* DH5α cells. The resulting plasmid was sequence verified by Sanger sequencing.

pNPTS138::mut35-3

To change the –35 promoter sequence of GTA cluster promoter from **AATCAT**C to Attgtcg , PCR amplifies fragments using NTO4184+NTO4504, NTO4185+NTO4505. PCR products were purified from gel and assembled to BamHI-HindIII-cut pNPTS138 using 2x Gibson mastermix following the manufacturer's instruction. The Gibson reaction was used to transform chemically competent *E. coli* DH5α cells. The resulting plasmid was sequence verified by Sanger sequencing.

pNTP2864::mut35-3

GTA cluster promoter with the –35 sequence changed from **AATCAT**C to Attgtcg was removed from pNPTS138::mut35-3 as EcoRI-KpnI fragment and ligated to EcoRI-KpnI-cut pNTP2864:: PgtaT (-153 to +229). The ligation mixture was used to transform chemically competent *E. coli* DH5α cells. The resulting plasmid was sequence verified by Sanger sequencing.

pNPTS138::PrsaA

PrsaA was amplified by PCR from *C. crescentus* genomic DNA using NTO4252+NTO4253. PCR products were purified from gel and assembled to BamHI-HindIII-cut pNPTS138 using 2x Gibson mastermix following the manufacturer's instruction. The Gibson reaction was used to transform chemically competent *E. coli* DH5α cells. The resulting plasmid was sequence verified by Sanger sequencing.

pNTP2864:: PrsaA

PrsaA was removed from pNPTS138:: PrsaA as EcoRI-KpnI fragment and ligated to EcoRI-KpnI-cut pNTP2864:: PgtaT (-153 to +229). The ligation mixture was used to transform chemically competent *E. coli* DH5α cells. The resulting plasmid was verified by restriction digestion.

pNPTS138::terGTA

Using genomic DNA as template, DNA fragments were amplified by PCR using NTO4094+NTO2930, NTO2931+NTO2935, NTO2936+NTO4095. PCR products were purified from gel and assembled to BamHI-HindIII-cut pNPTS138 using 2x Gibson mastermix following the manufacturer's instruction. The Gibson reaction was used to transform chemically competent *E. coli* DH5α cells. The resulting plasmid was sequence verified by whole-plasmid sequencing (Plasmidsaurus).

pNTP2884:: terGTA

terGTA was removed from pNPTS138::terGTA as BamHI-HindIII fragment and ligated to BamHI-HindIII cut pNTP2884. The ligation mixture was used to transform chemically competent *E. coli* DH5α cells. The resulting plasmid was verified by restriction digestion.

pNTP2864:: PgtaT (-153 to +229) + terGTA

terGTA was removed from pNPTS138::terGTA as a BamHI-HindIII fragment and ligated to BamHI-HindIII cut pNTP2864:: PgtaT (-153 to +229). The ligation mixture was used to transform chemically competent *E. coli* DH5α cells. The resulting plasmid was verified by whole-plasmid sequencing (Plasmidsaurus).

pNTP2864::PrsaA + terGTA

PrsaA promoter was removed from pNPTS138:: PrsaA as an EcoRI-KpnI fragment and ligated to EcoRI-KpnI-cut pNTP2864:: PgtaT (-153 to +229) + terGTA. The ligation mixture was used to transform chemically competent *E. coli* DH5α cells. The resulting plasmid was verified by Plasmidsaurus.

pNTP2884

pNTP2864:: PgtaT (-153 to +229) was cut with EcoRI and KpnI to remove the PgtaT-153 to +229, blunted with DNA polymerase I (NEB) and re-ligated using T4 ligase (NEB). The ligation mixture was used to transform chemically competent *E. coli* DH5α cells. The resulting plasmid was verified by Sanger sequencing.

pCOLA-Duet1::6xhis gafZ

gafZ was amplified by PCR from genomic DNA using NTO2693+NTO2694. PCR products were purified from gel and assembled to EcoRI-HindIII cut pCOLA-Duet1 using 2x Gibson mastermix following the manufacturer's instruction. The Gibson reaction was used to transform chemically competent *E. coli* DH5α cells. The resulting plasmid was sequence verified by Sanger sequencing.

pCOLA-Duet1::6xhis gafZ gafY

gafY was amplified by PCR from genomic DNA using NTO2691+NTO2692. PCR products were purified from gel and assembled to NdeI-KpnI cut pCOLA-Duet1::6xhis gafZ using 2x Gibson mastermix following the manufacturer's instruction. The Gibson reaction was used to transform chemically competent *E. coli* DH5α cells. The resulting plasmid was sequence verified by Sanger sequencing.

pCOLADuet-1::6xhis rpoD gafY

rpoD was amplified by PCR from genomic DNA using NTO4289+NTO4290. PCR products were purified from gel and assembled to EcoRI-HindIII cut pCOLA-Duet1::6xhis gafZ gafY using 2x Gibson mastermix following the manufacturer's instruction. The Gibson reaction was used to transform chemically competent *E. coli* DH5α cells. The resulting plasmid was sequence verified by Sanger sequencing.

pCOLADuet-1::6xhis rpoD (domain 3+4)

pCOLADuet-1::6xhis rpoD gafY was cut with NdeI and KpnI to remove the *gafY* insert. The backbone containing 6xhis rpoD was treated with Klenow to blunt the ends and ligated using T4 ligase. The ligation mixture was used to transform chemically competent *E. coli* DH5α cells. The resulting plasmid was purified and verified by Sanger sequencing. The plasmid was subsequently cut with EcoRI-HindIII to remove the *rpoD* insert. The newly generated backbone was ligated to EcoRI-HindIII cut rpoD3+4

fragment which was amplified by PCR using NTO4349+NTO4290. The ligation reaction was used to transform chemically competent *E. coli* DH5α cells. The resulting plasmid was sequence verified by Sanger sequencing.

pET15b::gafY

gafY orf was amplified by PCR using NTO4346 and NTO4347. PCR products were purified from gel and assembled to NcoI-BamHI-cut pET15b using 2x Gibson mastermix following the manufacturer's instruction. The Gibson reaction was used to transform chemically competent *E. coli* DH5α cells. The resulting plasmid was sequence verified by Sanger sequencing.

pNPTS138:: ΔgafZ

To delete *gafZ*, the flanking regions (~500bp) of the deletion site were amplified by PCR using NTO2634+NTO2635, NTO2636+NTO2637. PCR products were purified from gel and assembled to BamHI-HindIII-cut pNPTS138 using 2x Gibson mastermix following the manufacturer's instruction. The Gibson reaction was used to transform chemically competent *E. coli* DH5α cells. The resulting plasmid was sequence verified by Sanger sequencing, and introduced into *ΔlacA* cells to make NTS3086.

pNPTS138::ΔrogA

To delete *rogA*, the flanking regions (~500bp) of the deletion site were amplified by PCR using NTO2194+NTO2195, NTO2196+NTO2197. PCR products were purified from gel and assembled to BamHI-HindIII-cut pNPTS138 using 2x Gibson mastermix following the manufacturer's instruction. The Gibson reaction was used to transform chemically competent *E. coli* DH5α cells. The resulting plasmid was sequence verified by Sanger sequencing, and introduced into *ΔlacA* cells to make NTS3011, into NTS3086 to make NTS3124, and into *C. crescentus* CB15N to make NTS2240.

pNPTS138::terT1

terT1 was amplified by PCR from pBXMCS-2 using NTO4151 and NTO4152. PCR products were purified from gel and assembled to BamHI-HindIII-cut pNPTS138 using 2x Gibson mastermix following the manufacturer's instruction. The Gibson reaction was used to transform chemically competent *E. coli* DH5α cells. The resulting plasmid was sequence verified by Sanger sequencing.

pNTP2864:: PgtaT (-153 to +229) + terT1

terT1 was restricted out from pNPTS138::terT1 as a BamHI-HindIII-cut fragment and ligated to BamHI-HindIII-cut pNTP2864::PgtaT (-153 to +229). The ligation reaction was used to transform chemically competent *E. coli* DH5α cells. The resulting plasmid was sequence verified by Sanger sequencing.

pNTP2884:: terT1

pNTP2864:: PgtaT (-153 to +229) + terT1 was cut with EcoRI-KpnI to remove the PgtaT (-153 to +229), blunted with DNA polymerase I (NEB) and re-ligated using T4 ligase (NEB). The ligation mixture was used to transform chemically competent *E. coli* DH5α cells. The resulting plasmid was verified by Sanger sequencing.

pNTP2864::PrsaA + terT1

PrsaA was removed from pNPTS138::PrsaA as an EcoRI-KpnI fragment and ligated to EcoRI-KpnI-cut pNTP2864::PgtaT (-153 to +229) + terT1. The ligation reaction was used to transform chemically competent *E. coli* DH5α cells. The resulting plasmid was sequence verified by Sanger sequencing.

The *rogA*::tet^R allele from NTS2275 was introduced by ΦCr30-mediated transduction to generate Δ*rogA::tetracyclineR* containing mutants.

The *spoT*::tet^R allele from NTS2374 was introduced by ΦCr30-mediated transduction into NTS2240 to generate NTS2685

SUPPLEMENTARY REFERENCES

1. Arellano, B. H., Ortiz, J. D., Manzano, J. & Chen, J. C. Identification of a dehydrogenase required for lactose metabolism in Caulobacter crescentus. *Appl. Environ. Microbiol.* **76**, 3004–3014 (2010).
2. Thanbichler, M., Iniesta, A. A. & Shapiro, L. A comprehensive set of plasmids for vanillate- and xylose-inducible gene expression in Caulobacter crescentus. *Nucl. Acids Res.* **35**, e137–e137 (2007).
3. Tran, N. T. *et al.* Permissive zones for the centromere-binding protein ParB on the Caulobacter crescentus chromosome. *Nucleic Acids Res* **46**, 1196–1209 (2018).

4. Gozzi, K., Tran, N. T., Modell, J. W., Le, T. B. K. & Laub, M. T. Prophage-like gene transfer agents promote Caulobacter crescentus survival and DNA repair during stationary phase. *PLOS Biology* **20**, e3001790 (2022).
