# Supplementary material for: Control of a gene transfer agent cluster in Caulobacter crescentus by transcriptional activation and anti-termination
Source: Nat Commun. 2024 Jun 4;15:4749. doi: 10.1038/s41467-024-49114-2 (PMC11150451; doi:10.1038/s41467-024-49114-2)
Supplement: Supplementary file 3 — Description of Additional Supplementary Files [file 41467_2024_49114_MOESM3_ESM.pdf]

Description of Additional Supplementary Files

File Name: Supplementary Data 1
Description: Coding sequences and their annotated functions on the main CcGTA cluster

File Name: Supplementary Data 2
Description: Strains, plasmids, and oligonucleotides in this study

File Name: Supplementary Data 3
Description: List of ChIP-seq data used in this study

File Name: Supplementary Data 4
Description: Statistics of MACS2-detected ChIP-seq peaks in this study
