# Supplementary material for: Control of a gene transfer agent cluster in Caulobacter crescentus by transcriptional activation and anti-termination
Source: Nat Commun. 2024 Jun 4;15:4749. doi: 10.1038/s41467-024-49114-2 (PMC11150451; doi:10.1038/s41467-024-49114-2)
Supplement: Supplementary file 8 — Reporting Summary [file 41467_2024_49114_MOESM8_ESM.pdf]

nature portfolio

Corresponding author(s): Dr. Tung Le

Last updated by author(s): 10/5/2024

Reporting Summary

Nature Portfolio wishes to improve the reproducibility of the work that we publish. This form provides structure for consistency and transparency in reporting. For further information on Nature Portfolio policies, see our Editorial Policies and the Editorial Policy Checklist.

Statistics

For all statistical analyses, confirm that the following items are present in the figure legend, table legend, main text, or Methods section.

n/a	Confirmed	
☐	☒	The exact sample size (*n*) for each experimental group/condition, given as a discrete number and unit of measurement
☐	☒	A statement on whether measurements were taken from distinct samples or whether the same sample was measured repeatedly
☐	☒	The statistical test(s) used AND whether they are one- or two-sided<br>*Only common tests should be described solely by name; describe more complex techniques in the Methods section.*
☒	☐	A description of all covariates tested
☒	☐	A description of any assumptions or corrections, such as tests of normality and adjustment for multiple comparisons
☐	☒	A full description of the statistical parameters including central tendency (e.g. means) or other basic estimates (e.g. regression coefficient) AND variation (e.g. standard deviation) or associated estimates of uncertainty (e.g. confidence intervals)
☐	☒	For null hypothesis testing, the test statistic (e.g. *F*, *t*, *r*) with confidence intervals, effect sizes, degrees of freedom and *P* value noted<br>*Give P values as exact values whenever suitable.*
☒	☐	For Bayesian analysis, information on the choice of priors and Markov chain Monte Carlo settings
☒	☐	For hierarchical and complex designs, identification of the appropriate level for tests and full reporting of outcomes
☒	☐	Estimates of effect sizes (e.g. Cohen's *d*, Pearson's *r*), indicating how they were calculated

Our web collection on statistics for biologists contains articles on many of the points above.

Software and code

Policy information about availability of computer code

Data collection	Amersham Imager 600 (GE Healthcare), BIORAD CFX96 software
Data analysis	Adobe Illustrator v. 28 (Adobe), Excel 365 (Microsoft), bowtie 1 (https://bowtie-bio.sourceforge.net/index.shtml), bedtools 2.17.0 (https://bedtools.readthedocs.io/en/latest/), samtools 0.1.19 (http://www.htslib.org/), MACS2 (https://pypi.org/project/MACS2/), R 3.2.4 (https://www.r-project.org/), and GraphPad Prism 10 (https://www.graphpad.com/)

For manuscripts utilizing custom algorithms or software that are central to the research but not yet described in published literature, software must be made available to editors and reviewers. We strongly encourage code deposition in a community repository (e.g. GitHub). See the Nature Portfolio guidelines for submitting code & software for further information.

Data

Policy information about availability of data

All manuscripts must include a data availability statement. This statement should provide the following information, where applicable:

- Accession codes, unique identifiers, or web links for publicly available datasets
- A description of any restrictions on data availability
- For clinical datasets or third party data, please ensure that the statement adheres to our policy

All relevant data are provided within the manuscript and supplementary files. ChIP-seq data have been uploaded to the GEO repository (accession code: GSE247216). Data in the GEO are fully accessible to the public domain and reviewers.

Research involving human participants, their data, or biological material

Policy information about studies with human participants or human data. See also policy information about sex, gender (identity/presentation), and sexual orientation and race, ethnicity and racism.

Reporting on sex and gender	NA
Reporting on race, ethnicity, or other socially relevant groupings	NA
Population characteristics	NA
Recruitment	NA
Ethics oversight	NA

Note that full information on the approval of the study protocol must also be provided in the manuscript.

Field-specific reporting

Please select the one below that is the best fit for your research. If you are not sure, read the appropriate sections before making your selection.

☒ Life sciences ☐ Behavioural & social sciences ☐ Ecological, evolutionary & environmental sciences

For a reference copy of the document with all sections, see nature.com/documents/nr-reporting-summary-flat.pdf

Life sciences study design

All studies must disclose on these points even when the disclosure is negative.

Sample size	No statistical test was used to determine sample size. The sample sizes are based on established protocols and the potential to provide reliable and representative data. Note that all experiments are in-bulk, population averaged of ~millions individual bacteria.
Data exclusions	No data were excluded from the analyses
Replication	We routinely analyzed multiple independent strains to verify the phenotypes observed. All experiments were performed at least twice to ensure reproducibility, and similar results were obtained throughout.
Randomization	Strains for different experiments were selected randomly for inoculation from plate. All strains were grown under similar conditions, hence are equivalent at the start of the experiment. Observed differences are due to the difference in genotype of analyzed strains in this study.
Blinding	This was not necessary for this in-bulk/population-averaged microbiology study. All strains were grown under similar conditions, hence are equivalent at the start of the experiment. Observed differences are due to the difference in genotype of analyzed strains in this study.

Reporting for specific materials, systems and methods

We require information from authors about some types of materials, experimental systems and methods used in many studies. Here, indicate whether each material, system or method listed is relevant to your study. If you are not sure if a list item applies to your research, read the appropriate section before selecting a response.

Materials & experimental systems

n/a	Involved in the study	
☐	☒	Antibodies
☒	☐	Eukaryotic cell lines
☒	☐	Palaeontology and archaeology
☒	☐	Animals and other organisms
☒	☐	Clinical data
☒	☐	Dual use research of concern
☒	☐	Plants

Methods

n/a	Involved in the study	
☐	☒	ChIP-seq
☒	☐	Flow cytometry
☒	☐	MRI-based neuroimaging

Antibodies

Antibodies used

anti-FLAG antibody (Sigma Aldrich, Cat# F7425-.2MG), anti-V-SVG antibody (Sigma Aldrich, Cat#A1970-1ML), anti-GafY polyclonal antibody (custom made, Biosynth Laboratories Limited UK), anti-CCNA03882 (GtaL) polyclonal antibody (custom made, Biosynth Laboratories Limited UK), anti-ParB polyclonal antibody (custom made, Biosynth Laboratories Limited UK). Dilution factor was reported in the Methods section of the manuscript.

Validation

The specificity of all antibodies, especially custom-made polyclonal antibodies, used in this study was verified against lysates from deletion mutant strains or non-tagged strains of Caulobacter crescentus. Validation of commercially available antibodies was based on technical data sheets from manufacturers.
anti-FLAG antibody (Sigma Aldrich, Cat# F7425-.2MG): https://www.sigmaaldrich.com/GB/en/product/sigma/f7425#product-documentation
anti-V-SVG antibody (Sigma Aldrich, Cat#A1970-1ML): https://www.sigmaaldrich.com/GB/en/product/sigma/a1970#product-documentation

Plants

Seed stocks

Report on the source of all seed stocks or other plant material used. If applicable, state the seed stock centre and catalogue number. If plant specimens were collected from the field, describe the collection location, date and sampling procedures.

Novel plant genotypes

Describe the methods by which all novel plant genotypes were produced. This includes those generated by transgenic approaches, gene editing, chemical/radiation-based mutagenesis and hybridization. For transgenic lines, describe the transformation method, the number of independent lines analyzed and the generation upon which experiments were performed. For gene-edited lines, describe the editor used, the endogenous sequence targeted for editing, the targeting guide RNA sequence (if applicable) and how the editor was applied.

Authentication

Describe any authentication procedures for each seed stock used or novel genotype generated. Describe any experiments used to assess the effect of a mutation and, where applicable, how potential secondary effects (e.g. second site T-DNA insertions, mosiacism, off-target gene editing) were examined.

ChIP-seq

Data deposition

☒ Confirm that both raw and final processed data have been deposited in a public database such as GEO.

☒ Confirm that you have deposited or provided access to graph files (e.g. BED files) for the called peaks.

Data access links
May remain private before publication.

https://www.ncbi.nlm.nih.gov/geo/query/acc.cgi?acc=GSE247216
Data are already fully open to the public domain

Files in database submission

See Supplementary table 3 for the list of ChIP-seq used in this study:

ChIP-seq samples Genetic backgrounds antibody replicates Source
sample 1 CB15N WT anti-FLAG replicate 1 This study
sample 2 CB15N WT anti-FLAG replicate 2 This study
sample 3 CB15N ihfB::ihfB-flag anti-FLAG replicate 1 This study
sample 4 CB15N ihfB::ihfB-flag anti-FLAG replicate 2 This study
sample 5 ΔrogA anti-FLAG replicate 1 This study
sample 6 ΔrogA anti-FLAG replicate 2 This study
sample 7 ΔrogA ihfB::ihfB-flag anti-FLAG replicate 1 This study
sample 8 ΔrogA ihfB::ihfB-flag anti-FLAG replicate 2 This study
sample 9 ihfA::ihfA-flag anti-FLAG replicate 1 This study
sample 10 ihfA::ihfA-flag anti-FLAG replicate 2 This study
sample 11 ΔrogA ihfA::ihfA-flag anti-FLAG replicate 1 This study
sample 12 ΔrogA ihfA::ihfA-flag anti-FLAG replicate 2 This study
sample 13 ΔrogA gtaT IBE (TT to GG) anti-FLAG replicate 1 This study
sample 14 ΔrogA gtaT IBE (TT to GG) anti-FLAG replicate 2 This study
sample 15 ΔrogA gafY IBE (TT to GG) anti-FLAG replicate 1 This study
sample 16 ΔrogA gafY IBE (TT to GG) anti-FLAG replicate 2 This study
sample 17 ΔrogA gtaT IBE (TT to GG) ihfB::ihfB-flag anti-FLAG replicate 1 This study
sample 18 ΔrogA gtaT IBE (TT to GG) ihfB::ihfB-flag anti-FLAG replicate 2 This study
sample 19 ΔrogA gafY IBE (TT to GG) ihfB::ihfB-flag anti-FLAG replicate 1 This study
sample 20 ΔrogA gafY IBE (TT to GG) ihfB::ihfB-flag anti-FLAG replicate 2 This study
sample 21 ΔrogA gtaT IBE2-3-4-5 (TT to GG) anti-FLAG replicate 1 This study
sample 22 ΔrogA gtaT IBE2-3-4-5 (TT to GG) anti-FLAG replicate 2 This study
sample 23 ΔrogA gtaT IBE2-3-4-5 (TT to GG) ihfB::ihfB-flag anti-FLAG replicate 1 This study
sample 24 ΔrogA gtaT IBE2-3-4-5 (TT to GG) ihfB::ihfB-flag anti-FLAG replicate 2 This study
sample 25 ΔrogA anti-GafY replicate 1 Gozzi et al (2022)
sample 26 ΔrogA anti-GafY replicate 2 Gozzi et al (2022)
sample 27 ΔrogA ΔgafY anti-GafY replicate 1 Gozzi et al (2022)

sample 28 ΔrogA ΔgafY anti-GafY replicate 2 Gozzi et al (2022)
sample 29 ΔrogA anti-FLAG replicate 1 Gozzi et al (2022)
sample 30 ΔrogA anti-FLAG replicate 2 Gozzi et al (2022)
sample 31 ΔrogA gafZ::gafZ-FLAG anti-FLAG replicate 1 Gozzi et al (2022)
sample 32 ΔrogA gafZ::gafZ-FLAG anti-FLAG replicate 2 Gozzi et al (2022)
sample 33 ΔrogA rpoC::rpoC-3xflag anti-FLAG replicate 1 This study
sample 34 ΔrogA rpoC::rpoC-3xflag anti-FLAG replicate 2 This study
sample 35 ΔrogA gafZ::gafZ-FLAG nusA::vsvg-nusA anti-VSVG replicate 1 This study
sample 36 ΔrogA gafZ::gafZ-FLAG nusG::nusG-vsvg anti-VSVG replicate 1 This study
sample 37 ΔrogA gafZ::gafZ-FLAG nusE::vsvg-nusE anti-VSVG replicate 1 This study
sample 38 ΔrogA gafZ::gafZ-FLAG anti-VSVG replicate 1 This study
sample 39 ΔrogA gafZ::gafZ-FLAG nusA::vsvg-nusA anti-VSVG replicate 2 This study
sample 40 ΔrogA gafZ::gafZ-FLAG nusG::nusG-vsvg anti-VSVG replicate 2 This study
sample 41 ΔrogA gafZ::gafZ-FLAG nusE::vsvg-nusE anti-VSVG replicate 2 This study
sample 42 ΔrogA gafZ::gafZ-FLAG anti-VSVG replicate 2 This study
sample 43 ΔrogA gafZ::gafZ-FLAG ZBE* anti-FLAG replicate 1 This study
sample 44 ΔrogA gafZ::gafZ-FLAG ZBE* anti-FLAG replicate 2 This study
sample 45 ΔrogA gafZ::gafZ-FLAG ZBE* anti-GafY replicate 1 This study
sample 46 ΔrogA gafZ::gafZ-FLAG ZBE* anti-GafY replicate 2 This study
sample 47 ΔrogA YBE* anti-GafY replicate 1 This study
sample 48 ΔrogA YBE* anti-GafY replicate 2 This study
sample 49 ΔrogA rpoD::flag-rpoD anti-FLAG replicate 1 This study
sample 50 ΔrogA rpoD::flag-rpoD anti-FLAG replicate 2 This study

Genome browser session
(e.g. UCSC)

Not applicable because there is no UCSC browser for the reference genome of a bacterium Caulobacter crescentus NA1000. However, all processed data have been uploaded to GEO and are available to the public, and actual ChIP-seq profiles are shown in the figures in the manuscript.

Methodology

Replicates

x2 biological replicates for each ChIP-seq experiment

Sequencing depth

2.3 to 12 million reads for each ChIP-seq experiment (on average 7.9 million reads per sample), single-end 50nt-75nt reads (see Supplementary Data 3)

Antibodies

anti-FLAG antibody (Sigma Aldrich, Cat# F7425-.2MG), anti-V-SVG antibody (Sigma Aldrich, Cat#A1970-1ML), anti-GafY polyclonal antibody (custom made, Biosynth Laboratories Limited UK), anti-CCNA03882 (GtaL) polyclonal antibody (custom made, Biosynth Laboratories Limited UK)

Peak calling parameters

MACS2 program was employed for peak calling, using the following command for example:
macs2 callpeak -t ./IHF_exp/output.sorted.bam -c ./IHF_control/output.sorted.bam -f BAM -g 4e+6 -m 2,30 -n IHFexpvscontrol
macs2 callpeak -t ./IHF_exp/output.sorted.bam -c ./IHF_control/output.sorted.bam -f BAM -g 4e+6 --nomodel -n IHFexpvscontrol

Data quality

2.3 to 12 million reads were used for each ChIP-seq experiments (on average 7.9 million reads per sample) (see Supplementary Table S3). We also only consider MACS2-called peaks with fold enrichment > 4 (for narrow peaks) and >2 (for broad peaks) and -log10(FDR q value) > 200 in both replicates. And peaks described in the manuscripts were also inspected visually in both replicates. ChIP-seq peaks above IBE 4 and 5 were not reliably detected by MACS2 but have recognizable IHF-binding motif and were further characterized in Supplementary Fig. 2.

Software

For analysis of ChIP-seq data, Hiseq 2500 or NextSeq 550 Illumina short reads (50 bp/75 bp) were mapped back to the C. crescentus NA1000 reference genome (NCBI Reference Sequence: NC-011916.1) or appropriate reference genomes with mutations at the IBE/YBE/ZBE, using Bowtie 1 and the following command: bowtie -m 1 -n 1 –best –strata -p 4 –chunkmbs 512 NA1000-bowtie –sam *.fastq > output.sam. Subsequently, the sequencing coverage at each nucleotide position was computed using BEDTools using the following command: bedtools genomecov -d -ibam output.sorted.bam -g NA1000.fna > coverage_output.txt. When necessary, MACS2 were employed to call peaks. Finally, ChIP-seq profiles were plotted with the x-axis representing genomic positions and the y-axis is the number of reads per base pair per million mapped reads (RPBPM) or number of reads per kb per million mapped reads (RPKPM) using custom R scripts. For the list of ChIP-seq datasets in this study, see Supplementary Data 3.
